# Natural hybridization and asymmetric introgression at the distribution margin of two *Buddleja* species with a large overlap

**DOI:** 10.1186/s12870-015-0539-9

**Published:** 2015-06-18

**Authors:** Rong-Li Liao, Yong-Peng Ma, Wei-Chang Gong, Gao Chen, Wei-Bang Sun, Ren-Chao Zhou, Tobias Marczewski

**Affiliations:** Kunming Botanical Garden; Key Laboratory for Plant Diversity and Biogeography of East Asia, Kunming Institute of Botany, Chinese Academy of Sciences, Kunming, 650201 Yunnan China; University of Chinese Academy of Sciences, Beijing, 100049 China; College of Life Science and Technology, Honghe University, Mengzi, 661199 Yunnan China; State Key Laboratory of Biocontrol and Guangdong Provincial Key Laboratory of Plant Resources, Sun Yat-sen University, Guangzhou, 510275 China

**Keywords:** Asymmetric introgression, *Buddleja*, Hybridization, Nuclear genes, Reproductive isolation

## Abstract

**Background:**

Natural hybridization in plants is universal and plays an important role in evolution. Based on morphology it has been presumed that hybridization occurred in the genus *Buddleja*, though genetic studies confirming this assumption have not been conducted to date. The two species *B. crispa* and *B. officinalis* overlap in their distributions over a wide range in South-West China, and we aimed to provide genetic evidence for ongoing hybridization in this study.

**Results:**

We investigated the occurrence of hybrids between the two species at the southern-most edge of the distribution of *B. crispa* using five nuclear loci and pollination experiments. The genetic data suggest substantial differentiation between the two species as species-specific alleles are separated by at least 7–28 mutations. The natural hybrids found were nearly all F1s (21 of 23), but backcrosses were detected, and some individuals, morphologically indistinguishable from the parental species, showed introgression. Pollen viability test shows that the percentage of viable pollen grains was 50 ± 4 % for *B. crispa*, and 81 ± 2 % for *B. officinalis*. This difference is highly significant (t = 7.382, p < 0.0001). Hand cross-pollination experiments showed that *B. crispa* is not successful as pollen-parent, but *B. officinalis* is able to pollinate *B. crispa* to produce viable hybrid seed. Inter-specific seed-set is low (8 seeds per fruit, as opposed to about 65 for intra-specific pollinations), suggesting post-zygotic reproductive barriers. In addition, one of the reference populations also suggests a history of introgression at other localities.

**Conclusions:**

The occurrence of morphologically intermediate individuals between *B. crispa* and *B. officinalis* at Xishan Mountain is unequivocally linked to hybridization and almost all examined individuals of the putative hybrids were likely F1s. Despite pollination experiments indicating higher chances for introgression into *B. officinalis* (hybrids only produced viable seed when crossed with *B. officinalis*), observed introgression was asymmetrical into *B. crispa.* This could be due to seeds produced by hybrids not contributing to seedlings, or other factors favoring the establishment of backcrosses towards *B. crispa*. However, further research will be needed to confirm these observations, as the small number of plants used for the pollination experiments could have introduced an artifact, for example if used individuals were more or less compatible than the species average, and also the small number of loci used could convey a picture of introgression that is not representative for the whole genome.

**Electronic supplementary material:**

The online version of this article (doi:10.1186/s12870-015-0539-9) contains supplementary material, which is available to authorized users.

## Background

Natural hybridization is ubiquitous in plants and has several evolutionary consequences including the origin and/or transfer of genetic adaptations, the origin of new ecotypes or species, and the reinforcement or break-down of reproductive barriers [1–5]. For closely related species with sympatric distribution, the formation and maintenance of reproductive isolating barriers is an important issue in speciation [6–8]. In such cases, species boundaries could be maintained by the elimination of intermediate hybrids due to low F_1_ fertility or hybrid breakdown [7, 9], or by F_1_ dominated hybrid zones, in which F_1_s exhibit apparent habitat-mediated superiority over other hybrid classes [10]. The latter is an extreme case scenario, and even a small number of hybrids beyond the F1 generation might be enough to provide a genetic bridge enabling introgression [10, 11].

*Buddleja crispa* Benth. and *B. officinalis* Maxim are two species in the family Scrophulariaceae, both having the habit of shrubs or, in the case of *B. officinalis*, rarely small trees, reaching a height of about 3 m. *B. crispa* grows mostly at altitudes of above 2000 m, and prefers exposed rocky habitats and dry river valleys [12]; this species has a distribution ranging from Afghanistan into the eastern Himalayas, where it reaches into the higher parts of the Himalayan foothills of South-West China in Yunnan and Sichuan. The distribution of *B. officinalis* is restricted to comparably lower altitudes in South to South-west China, but it has a large overlap with *B. crispa* in the foothills, where both altitudinal ranges meet at an approximate altitude of 1500 – 2500 m; *B. officinalis* prefers forest edges on mountains, and thickets on riverbanks. Both species are predominantly outcrossing, though partial self-fertility and autogamy were occasionally observed in *B. crispa* [13]*.* Both species are diploid with chromosome numbers 2 *n* = 38, and have an intra-specifically variable morphology, including flower color, leaf size and shape, and indumentum thickness. Furthermore, both species flower in early spring (*B. crispa* – March to April; *B. officinalis* – February to May [14]; and are likely to share pollinators, mostly butterflies [15–17]; however, despite the extensive overlap, no hybrids between the species have been reported to date. When the two species grow sympatrically, *B. crispa* is mostly found at the higher altitudes, often growing under extreme conditions on sheer rock faces, mostly with very little soil available, and very exposed. In this habitat the plants frequently remain rather small, not exceeding 1 m in height. *B. officinalis* is never found growing under these extreme conditions, and mostly replaces *B. crispa* at lower altitudes, where it is often growing amongst other vegetation below the canopy of larger trees, or on disturbed ground, but always with a considerable layer of soil available. Hence the two species seem to have different ecological requirements in sympatry, but *B. crispa* does grow in *B. officinalis* habitat, when the species is not present, and at sympatric sites the species are in close contact due to the proximity of respectively suitable sites (e.g. exposed rocks and cliffs amidst a forest covered slope). Although no hybrids between the species have been reported, numerous intermediate individuals have been discovered at the south-eastern most range limit of *B. crispa*, namely Xishan Mountain near Kunming city in Yunnan (Sun Weibang, personal observation).

Geological records suggest profound climatic changes in the region over the last 30,000 years leading to three major changes in floral composition, with the last having occurred 13,000 years ago [18]. Additionally, temperature changes led to significant forest range changes, and changes in species abundance up to about 2500 to 1500 years ago [19]. It is likely, that these environmental changes will also have resulted in range contraction and expansion of *B. crispa* and *B. officinalis*. The geographical location of the potential hybrid zone is especially interesting, as it allows investigating questions about reproductive isolation between species at the extremes of their distribution range. In the present study, our main aims are to test the hybridization hypothesis, and to investigate reproductive isolation barriers in this special location at the distribution margin of one of the two species. The specific questions we want to ask are: (1) Are these morphologically intermediate individuals really hybrids between *B. crispa* and *B. officinalis*? (2) What is the composition of the hybrid zone on Xishan Mountain? Is there any introgression between the two species? and (3) Is there any evidence for reproductive barriers between these two species?

## Results

### Morphological analysis for *B. officinalis*, *B. crispa* and their putative hybrid

**Fig. 1:**
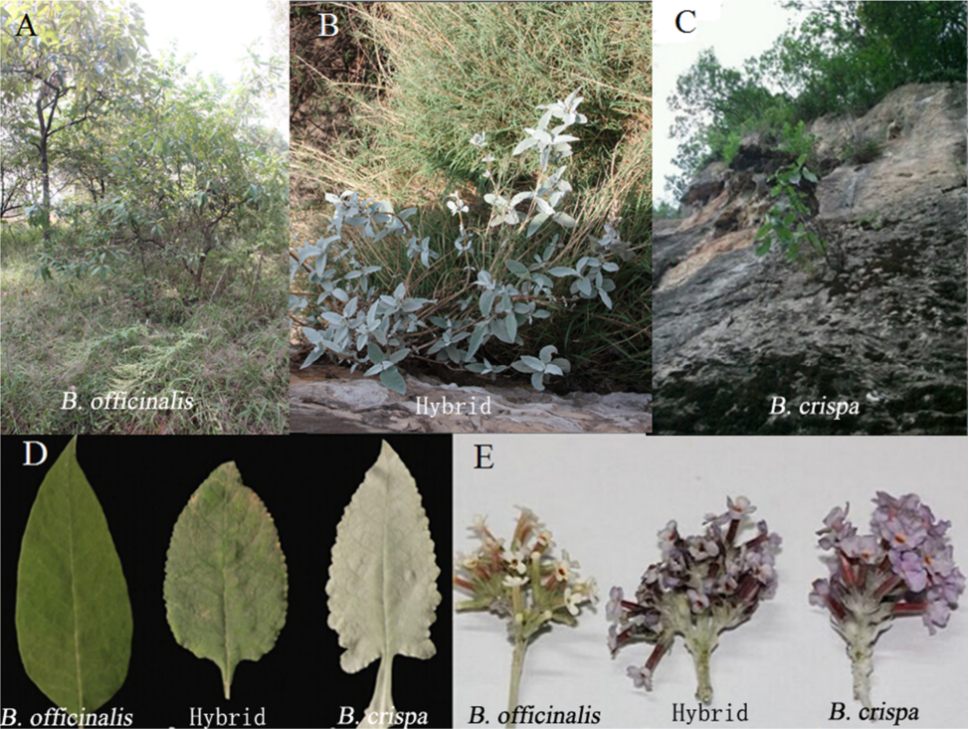
Habitats (**a, b, c**), leaf (**d**) and flower characteristics (**e**) of *B. officinalis*, *B. crispa* and the putative hybrids

At all sampling sites, *B. officinalis* and *B. crispa* could easily be distinguished using the four morphological characters: leaf shape, leaf margin, leaf base including petiole, and indumentum on the adaxial leaf surface (Fig. 1). Putative hybrid individuals had morphological characters intermediate between *B. crispa* and *B. officinalis*, although leaves are often distinct in shape from both of the parents, as the widest part of the leaf lamina is more central, and hence conforming to an elliptic shape. The Welch statistic calculated for the three *Buddleja* taxa we examined indicated significant differences for the leaf width (*F* = 51.236, *P* < 0.001). The leaf width of *B. officinalis* was smaller than that of both *B. crispa* and the putative hybrids (*P* < 0.001 for each comparison); however, there was no significant difference between *B. crispa* and the putative hybrid (*P* = 0.312) (Table 1).

**Table 1:** Morphological traits used to distinguish *B. officinalis*, *B. crispa* and their putative hybrid

Character	*B. officinalis*	*B. crispa*	Putative hybrid	F	Welch	*P* value
Leaf shape	Narrowly ovate	Ovate	Ovate-elliptic			
Leaf margin	Entire	Crenate	Sinuate			
Adaxial leaf surface indumentum	Glabrescent	Densely tomentose	Puberulent			
Leaf base including petiole	Cuneate, free	Auriculate, winged	Cuneate to decurrent			
Corolla color	Yellow-white	Lilac to purple	Lilac			
*L* (cm)	13.35 ± 1.69	13.54 ± 2.41	13.89 ± 1.90	0.563		0.572
*W* (cm)	4.99 ± 0.73^a^	7.35 ± 1.36^b^	6.85 ± 1.07^b^		51.236	<0.001
*L*/*W*	2.70 ± 0.36^a^	1.86 ± 0.30^b^	2.04 ± 0.14^c^		52.207	<0.001

mean ± standard deviation are shown for the three traits

*L* leaf length, *W* leaf width, *L*/*W* ratio of leaf length to leaf width

^a, b, c^:the means with different superscripts are significantly different from each other at the 0.05 level and based on Tamhane’s T2 test

While the leaf length was not significantly different between the three taxa (*F* = 0.563, *P* = 0.572, Table 1), ratios of leaf length to leaf width were significantly different between all three taxa (*F* = 52.207, *P* < 0.001). *B. officinalis* and *B. crispa* had the greatest and smallest ratios of leaf length to leaf width, respectively, with the putative hybrid being intermediate (Table 1).

### Pollen viability test and hand pollination experiments

In total 714 pollen grains of *B. crispa* and 445 pollen grains of *B. officinalis* were examined for viability. Based on staining with MTT, the percentage of viable pollen grains in just dehisced anthers was 50 ± 4 % for *B. crispa*, and 81 ± 2 % for *B. officinalis*. This difference is highly significant (*t* = 7.382, *p* < 0.0001).

Cross-pollination treatments showed a wide range of fruit set, ranging from 6 % (*B. officinalis*♀ × *B. crispa*♂) to 84 % (putative hybrid♀ × *B. officinalis*♂) (Table 2). However, the number of seeds per fruit was one order of magnitude higher for the intraspecific crosses *B. officinalis* (♀) × *B. officinalis* (♂) and *B. crispa* (♀) × *B. crispa* (♂) (~65 compared to 0.43-8, Table 2), resulting in many more viable seeds for these crosses, even with lower fruit set. In interspecific crosses there was a marked difference between the two species with regards to success as pollen donor and pollen recipient. While crosses with *B. crispa* as maternal parent always produced at least some viable seed, no viable seeds were produced in any heterospecific cross with *B. crispa* as paternal parent. *B. officinalis* on the other hand was always a successful pollen donor (Table 2).

**Table 2:** Fruit set, seed number, seed viability and number of viable seeds per fruit for nine pollination combinations among *B. officinalis*, *B. crispa* and their putative hybrid

Pollen recipient (♀)	Pollen donor (♂)	Number of flowers	Number of fruits	Fruit set (%)	Mean number of seeds per fruit	Seed viability (%)	Mean number of viable seeds per fruit^a^
*B. crispa*	*B. crispa*	20	11	55	65.50	34	22.27
	*B. officinalis*	24	14	58	8.00	68	5.44
	Putative hybrid	27	15	55	2.20	70	1.54
*B. officinalis*	*B. crispa*	32	2	6	1.00	0	0.00
	*B. officinalis*	23	10	43	65.00	91	59.15
	Putative hybrid	37	18	48	1.94	57	1.11
Putative hybrid	*B. crispa*	31	19	61	2.79	0	0.00
	*B. officinalis*	38	32	84	4.63	53	2.45
	Putative hybrid	45	7	15	0.43	0	0.00

^a^viable seeds per fruit = mean seed number × seed viablility

### Sequence analysis of four low-copy nuclear genes and nrETS

#### GapC1

**Fig. 2:**
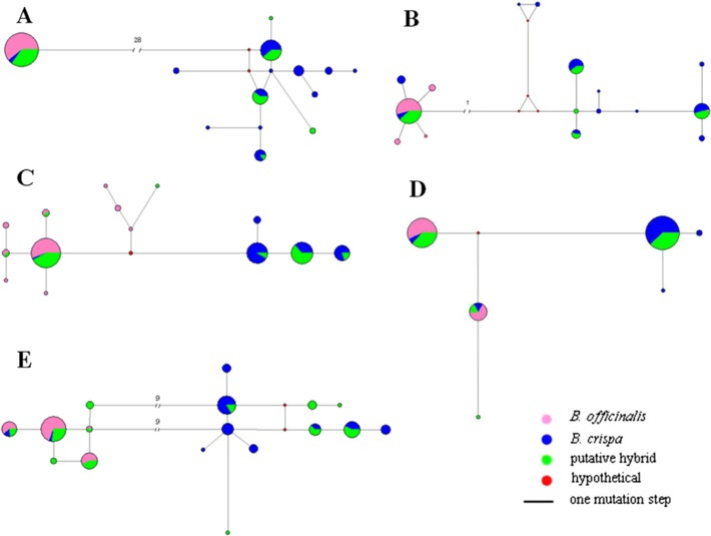
Haplotype networks for gapC1 (**a**), gapC2 (**b**), defA (**c**), fl1 (**d**) and nrETS (**e**). Pink, blue and green circles represent haplotypes of *B. officinalis*, *B. crispa* and their putative hybrid. Small red circles represent hypothetical or unsampled haplotypes. The number of mutations separating two haplotypes is indicated by the length of the connecting lines unless the number was shown on the line

The alignment of gapC1 spanned 608 bp, including only one 1-bp indel, which distinguished *B. officinalis* alleles from *B. crispa* alleles. Haplotype network analysis identified two highly divergent clusters separated by 28 nucleotide substitutions (Fig. 2a). Only one haplotype was present in *B. officinalis* individuals, which represents one of the clusters, while haplotypes present in *B. crispa* generally conform to the other; the only exception to this pattern are three individuals of *B. crispa* (Z13, Z15 and Z22) which had the haplotype found in *B. officinalis*. With regards to the putative hybrid individuals, all but two (P18 and P20) had two divergent haplotypes, each originating from one of the diverged clusters. The two individuals (P18 and P20) were homozygous at this locus, with P18 having the same sequence as *B. officinalis* and P20 possessing a unique haplotype nested within the *B. crispa* cluster.

#### GapC2

The alignment of gapC2 spanned 596 bp including one 1-bp indel distinguishing *B. officinalis* alleles from *B. crispa* alleles. Haplotype network analysis identified two major clusters separated by 7 nucleotide substitutions (Fig. 2b). One cluster comprised 4 out of 5 haplotypes of *B. officinalis,* the other8 out of 10 haplotypes of *B. crispa*. Additionally, two haplotypes from seven individuals of *B. crispa* (Z6, Z13, Z14, Z15, Z17, Z18 and Z22) were nested within the *B. officinalis* cluster, and one haplotype from one individual of *B. officinalis* (M2) was nested within the *B. crispa* cluster.

Of the putative hybrid individuals, all but three (P13, P18 and P20) had two divergent haplotypes, one nested within each of the two divergent clusters. Two individuals (P18 and P20) were homozygous at this locus, having haplotypes from the *B. officinalis* and *B. crispa* clusters, respectively. Individual P13 had two haplotypes found in the *B. crispa* cluster.

#### DefA

The length of the sequenced fragment of *defA* was 349 bp for all individuals. Haplotype network analysis identified two clusters separated by four nucleotide substitutions (Fig. 2c). All 9 haplotypes of *B. officinalis* belonged to one of these clusters, while the other cluster contained 4 of the 5 haplotypes found in *B. crispa*. The remaining haplotype found in one individual of *B. crispa* (Z22) was identical to the major haplotype of *B. officinalis*. Of the putative hybrid individuals all but one (P18) had two divergent haplotypes, one from each of the two clusters. Individual P18 was homozygous at this locus for a *B. officinalis* haplotype.

#### Fl1

The alignment of *fl1* spanned 270 bp including one 3-bp indel distinguishing *B. officinalis* and *B. crispa* alleles*.* As in the cases above the two clusters identified employing haplotype network analysis corresponded largely to haplotypes found in *B. officinalis* and *B. crispa*, respectively. The clusters were separated by 6 nucleotide substitutions (Fig. 2d), and *B. officinalis* haplotypes grouped exclusively in one cluster. While 3 of 5 haplotypes of *B. crispa* were in the other cluster, 2 haplotypes from six individuals of *B. crispa* (Z9, Z13, Z15, Z20, Z21 and Z22) clustered with *B. officinalis* haplotypes. With the exception of P20, all of the putative hybrid individuals had two divergent haplotypes, one from each of the two clusters; individual P20 was homozygous for a *B. crispa* haplotype at this locus.

#### NrETS

The length of the sequenced fragment of the nrETS region was 337 bp in all individuals. Haplotype network analysis identified two clusters separated by 9 nucleotide substitutions (Fig. 2e). All four haplotypes of *B. officinalis* were grouped in one cluster, and 8 of 10 haplotypes of *B. crispa* were grouped in the other cluster. Interestingly, 2 haplotypes from three individuals of *B. crispa* (Z13, Z15 and Z22) clustered with *B. officinalis*. The putative hybrid individuals showed the same pattern as before, all but one individual (P18) having two divergent haplotypes, one from each of the two clusters; individual P18 was homozygous for a *B. officinalis* haplotype.

### NewHybrids analysis

**Fig. 3:**
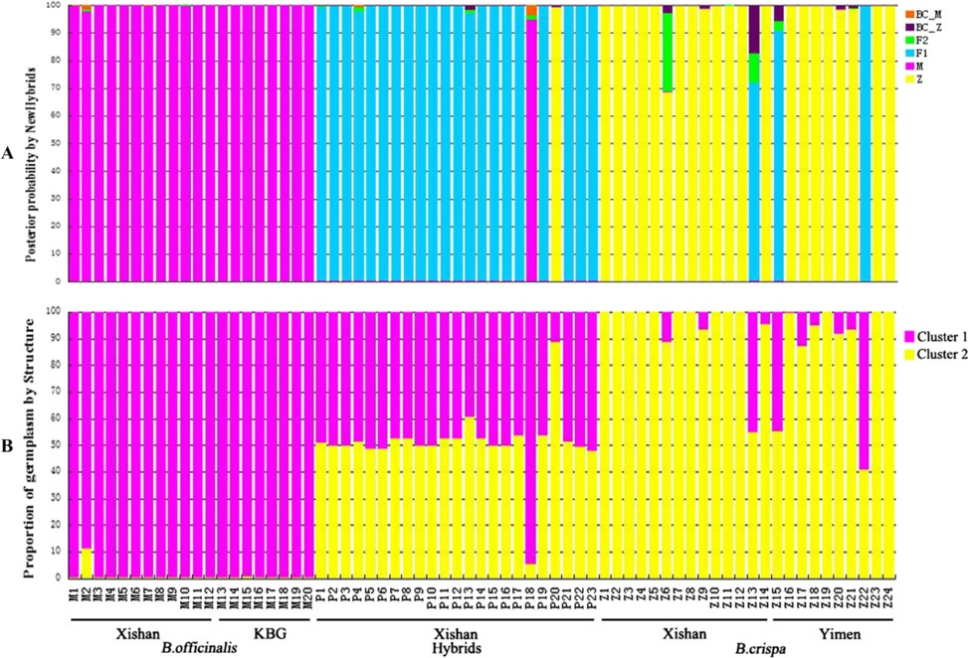
Genotype class assignment by NewHybrids (**a**) and clustering analysis by STRUCTURE (**b**) for *B. officinalis*, *B. crispa* and putative hybrid individuals, based on sequence data of four low-copy nuclear genes and the nrETS region

Posterior probabilities for the assignment of individuals to certain genotype classes (parent, F1, F2, backcross) were obtained with the program NewHybrids. Individuals previously identified as *B. officinalis* based on morphological characters, were all assigned to *B. officinalis* with high posterior probabilities (>0.977). Of the 24 individuals morphologically identified as *B. crispa*, 20 individuals were assigned to *B. crispa* with high posterior probabilities (>0.982), but 3 individuals were assigned to the F1 class (Fig. 3a - Z13, Z15 and Z22) and one to *B. crispa* with much lower probability (Z6). Of the 23 individuals morphologically identified as putative hybrids, 21 individuals were assigned to the F_1_ class with high posterior probabilities (>0.969); two individuals, however, were classed as *B. officinalis* (P18, 0.949) and *B. crispa* (P20, 0.991), respectively (Fig. 3a; Additional file 1: Table S1).

### Structure analysis

The most likely number of clusters (K) for the whole dataset, as determined by the highest ΔK [20], was chosen as the true value of K. The Structure analysis for the three taxa yielded a highest ΔK value for K = 2 (Additional file 2: Figure S1), indicating that two genetic clusters were sufficient to explain structures observed in the three groups. When K = 2, alleles of individuals morphologically identified as *B. officinalis* mostly originate from one cluster (q = 0.991 ± 0.024), whereas alleles from 21 of 24 individuals morphologically identified as *B. crispa* originate mostly from the other (q = 0.910 ± 0.168). Therefore, the two clusters were interpreted as corresponding to *B. officinalis* and *B. crispa*, respectively. The same three individuals with *B. crispa* morphology that were classed as F1s by NewHybrids had alleles derived from both clusters in about equal proportion (Fig. 3b; Additional file 1: Table S2, Z13, Z15 and Z22; q_cluster 2 = 0.548, 0.550 and 0.406, respectively).

Nearly all of the putative hybrid individuals, 21 out of 23, had alleles in equal proportion from both clusters (q was about 0.479 to 0.607). Individuals P18 and P20 had a considerably higher proportion of alleles from one cluster than the other (q_cluster 1 = 0.947, q_cluster 2 = 0.887, respectively, Additional file 1: Table S2). Lastly, one of the individuals with *B. officinalis* morphology (M2) shows low admixture from the *B. crispa* cluster (q_cluster 2 = 0.110, Additional file 1: Table S2).

## Discussion

### Hybridization between *B. officinalis* and *B. crispa* on Xishan Mountain

Both species investigated in this study have a variable phenotype with regards to many morphological characters. Therefore, one major objective of this preliminary study is to ascertain that the apparent morphological intermediacy has indeed resulted from hybridization, rather than extraordinary variability of one of the species in this area, because morphological intermediacy is not invariably associated with hybrids [21]. As can be seen from the haplotype network analysis (Fig. 2), the two species are considerably differentiated, with one of the loci showing as many as 28 substitutions between alleles found in the two species (Fig. 2a). This large differentiation between the species makes the genetic identification of hybrids less ambiguous, and therefore, the heterozygote state of all morphologically intermediate plants at most loci gives strong evidence that these individuals are indeed hybrids of *B. crispa* and *B. officinalis*.

That these two species can successfully form hybrids is additionally supported by the pollination experiments. While *B. crispa* was not successful as a pollen donor for *B. officinalis*, viable seeds produced by *B. crispa* when receiving pollen from *B. officinalis* were as much as 24 % of conspecific viable seed set (5.44 / 22.27, Table 2), indicating that sufficient hybrid seeds can be produced in the wild to allow the establishment of hybrids under suitable conditions. Furthermore, *B. crispa* produces an exceptionally low percentage of viable seeds from conspecific pollinations when compared to conspecific pollination in *B. officinalis* (34 % opposed to 91 %, Table 2). One explanation for the low seed production could be that the pollination experiments were carried out on transplanted plants, introduced to KBG, and that conditions at KBG are reflecting the ecological requirements of *B. officinalis* much better than those of *B. crispa*, hence *B. crispa* plants might have suffered substantial stress against the seed production. It would therefore have been desirable to perform the pollination experiments in the wild population at Xishan. However, this was not possible due to lack of permission from the local authorities, and the interference of the frequenting public. Another explanation could be the significantly different pollen viabilities of the two species (*B. crispa* 50 % vs. *B. officinalis* 81 %). However, it would be expected that insufficient viable pollen would foremost affect the number of seeds produced, not the viability of produced seeds. As the number of successfully fertilized flowers (55 %, 43 %; Table 2) and seeds per fruit (both ~65, Table 2) is nearly equal for both species, it seems unlikely that pollen viability had a significant effect.

### Hybrid zone composition and introgression

Despite the relatively low number of fertile hybrid seeds, as compared to seeds resulting from conspecific pollination (5.44 vs 22.27, Table 2), hybrids are frequent in the investigated area. Most of these hybrids are most likely to be F1s as identified by the NewHybrids analysis, and supported by their heterozygous state, always one allele each from each species, for all investigated loci. Hybrid zones which comprise prevalently F1 individuals have been reported before [10, 22, 23], and it has been hypothesized that a high frequency of F1 individuals can under certain circumstances impede interspecific gene flow effectively. For example in *Rhododendron* and *Encelia*, F1 individuals effectively outcompete all other hybrid classes, thereby impeding backcrossing and thus introgression [10, 11]. Under such circumstances the reproductive barrier seems to be mostly ecological as the parental species interbreed freely, and the F1 hybrids are highly fertile. The pollination experiments, however, point to a larger role of intrinsic incompatibilities, as opposed to ecological selection against hybrids, as the viable seed set for interspecific pollinations is lower than for intraspecific pollinations (Table 2). Furthermore, although most hybrids are F1s, some later generation hybrids were identified in the hybrid zone indicative of at least some successful backcrossing; two individuals of the morphologically intermediate individuals showed admixture with much higher contribution from one of the two species than would be expected for first generation hybrids (P18 and P20, Fig. 3). Additionally, several of the *B. crispa* individuals showed a low fraction of alleles derived from *B. officinalis* (Z6, Z13, Z15, Z17, Z22, Fig. 3). This relatively high number of *B. officinalis* alleles in a *B. crispa* background hints towards asymmetric introgression into this species. Due to factors such as phenology, gametopytic-sporophytic interactions during fertilization or organelle-nuclear gene interactions, asymmetric barriers in plants are quite common [24, 25], however, to elucidate which factors are most important for the present case requires further research.

Judging from the pollination experiments, a low number of backcrosses is expected due to the fact that the number of viable seeds produced for those crosses is relatively low (BC *officinalis* 1.11 and 2.45; BC *crispa* 1.54 and 0, Table 2). Additionally, pure *B. officinalis* and pure *B. crispa* individuals are more abundant in the population, making intraspecific pollinations, and interspecific pollinations between the parents, resulting in F1 offspring, more likely. Hence the occurrence of only few backcrosses can be expected, and their presence indicates that ecological selection is not strong enough to completely impede gene flow between the two species. Furthermore, from the individuals comprising the population at Xishan, one exhibiting pure *B. officinalis* morphology had a *B. crispa* allele at the *gapC2* locus (M02), and four individuals with *B. crispa* morphology (Z06, Z09, Z13, and Z14) showed different levels of admixture (Fig. 3), suggesting occasional backcrossing, and thus the possibility of introgression. Interestingly the data from the pollination experiments suggests that theoretically, assuming conditions only taking the production of viable seeds into account, more backcrosses towards *B. officinalis* could be expected. Generally seed set was low when a hybrid individual was used as one parent, but crosses with *B. officinalis* produced some viable seeds in each direction, while crosses with *B. crispa* were only successful with the hybrid as pollen donor (Table 2), suggesting that more backcross seed involving *B. officinalis* should be produced. Due to the small sample size it is possible that this is an artifact, but it is also possible that certain other factors favor backcrossing to *B. crispa* in the wild. It is widely accepted that many types of pre- and post- zygotic barriers can act together to prevent hybridization and introgression [26–28]. Artificial pollination experiments only cover a small subset of these barriers. For instance temporally variable barriers such as flowering period, pollinator preference and seedling establishment of hybrids in nature are very difficult to assess, and might have lead to a higher occurrence of backcrosses towards *B. crispa*. However, at least during the seedling stage ecological selection is likely to affect successful establishment, and with the present data adaptively favored introgression, mostly benefiting individuals with *B. crispa* background can not yet be ruled out.

Because hybrids had never before been reported for this species pair, and additionally during extensive fieldwork throughout the distribution range of *B. crispa*, hybrids had never been observed, the reference population for *B. crispa* was sampled relatively close to Xishan Mountain, as we intended to avoid large allelic differences due to distance between sampled populations. The reference site, Yimen, was therefore also situated at the southern extreme of the distribution of *B. crispa.* The Structure analysis indicated that several *B. crispa* individuals in this population show admixture from *B. officinalis* (Z15, Z17, Z18, Z20-22; Fig. 3), giving evidence that hybridization is not restricted to Xishan Mountain, and that introgression also occurred in this population. Due to the distinctiveness of the *B. crispa* and *B. officinalis* alleles it is unlikely that this pattern could have been caused by ancient shared alleles, and a more in-depth search around the sample site revealed several morphologically intermediate individuals. As the population was assumed to be pure, examination during the first collection was not thorough, and likely the genetically admixed individuals would have been identifiable by means of morphology.

Some of the admixed individuals were morphologically not distinguishable from pure individuals of one or the other of the parental species. Therefore, a more comprehensive sampling approach will be needed in future studies to investigate potential past admixture in areas where the species distributions overlap. We are not aware of other publications demonstrating hybridization between the two species, and previous observations made during fieldwork, covering a wide range of the overlap of the two species [17] (Sun Weibang, Chen Gao personal observation), did not hint towards hybridization at other localities, hence the present data suggest a difference in reproductive isolation between the two species at the southern edge of the distribution of *B. crispa* as opposed to the rest of the distribution range. If *B. crispa* is advancing its range southwards, this might be expected, as according to theory introgression of local genes will often accompany a range expansion [29]. If some of the introgressed alleles are, however, adaptive remains unclear.

## Conclusions

The occurrence of morphologically intermediate individuals between *B. crispa* and *B. officinalis* at Xishan Mountain is unequivocally linked to hybridization. Morphologically intermediate individuals were almost all F1s, but some individuals which were classed as one of the parental species seem to be backcrosses, or show introgression. The two species can produce viable hybrid seed under controlled conditions, and backcrossing in both directions is theoretically possible. Later generation backcrosses and introgression were detected at both *B. crispa* sample sites, and the data suggests gene flow in both directions, as one individual identified as *B. officinalis* showed low amounts of admixture originating from the *B. crispa* cluster. Furthermore, at least at Xishan Mountain there is evidence that this introgression is mostly asymmetric, as a substantially higher proportion of *B. officinalis* alleles was detected in *B. crispa* than *B. crispa* alleles in *B. officinalis*.

## Methods

### Plant sampling for molecular analysis

**Fig. 4:**
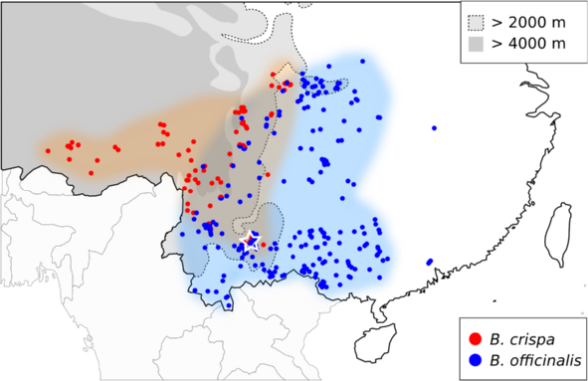
Geographical distribution of *B. officinalis* (blue) and *B. crispa* (red) in China, based on locality information of 710 specimens (474 *B. officinalis* and 236 *B. crispa*); data for the specimens were obtained from the Chinese Virtual Herbarium (http://www.cvh.org.cn, accessed Aug 22, 2014), and all available specimens were included. *B. crispa* is predominantly found at altitudes >2000 m, while *B. officinalis* grows mostly at lower altitudes. The location of the study site is indicated with a star

Comprehensive field surveys involving *B. crispa* and *B. officinalis* have been performed in the last decade (Sun Weibang, personal observation). Although the two species are sympatric in some regions, only one putative hybrid zone has been identified on Xishan Mountain, Kunming, Yunnan, China (Fig. 4), where many individuals with intermediate morphology between *B. crispa* and *B. officinalis* were observed (Fig. 1). These individuals were hypothesized as natural hybrids, and mostly occurred along a main road in the scenic area of Xishan. *B. officinalis* individuals can be found throughout the area, and certainly more than 500 individuals can be found on Xishan; a population size estimate of *B. crispa* is more difficult, as the plants grow on sheer cliffs, but in the area more than 100 plants should be present. In this study, 24, 20 and 23 individuals of *B. crispa*, *B. officinalis* and their putative hybrid were collected respectively. All hybrids and some of the parents were collected at Xishan; additionally, further individuals of *B. crispa* and *B. officinalis* were collected at Yimen and Kunming Botanical Garden (KBG), respectively (Table 3). *B. crispa* and *B. officinalis* were identified according to the morphological descriptions in the Flora of China [12]. The eight individuals from Yimen, were collected without thorough checking for hybrid characters, as the population was assumed to be pure. Therefore it is possible that some of the later identified hybrids would have showed intermediate characters. Directly after collection, leaf material was transferred to zip-lock plastic bags containing silica gel.

**Table 3:** Sampling details of the three *Buddleja* taxa in this study

Taxon	Number of individuals sampled (Sample ID)		
	Xishan, Kunming	Yimen, Yuxi	Kunming Botanical Garden, Kunming
*B. officinalis*	12 (M01-M12)	0	8 (M13-M20)
*B. crispa*	14 (Z01-Z14)	10 (Z15-Z24)	0
Putative hybrid	23 (P01-P23)	0	0

### Measurements and data analysis of morphological traits

Three leaves from ten healthy individuals for each of the three taxa were sampled from Xishan Mountain and then taken to KBG for morphological measurements. These samples were collected independently from the molecular samples and any overlap between them would be coincidental. Six leaf characters were assessed as follows: leaf indumentum, leaf shape, leaf base, leaf margin, leaf length (L, from the tip of the leaf to the position where the petiole joins the lamina) and leaf width (W, width at widest point). Additionally the flower color was noted for each of the individuals. Traits were analyzed with one-way ANOVA, where specie was treated as a fixed factor. When variances within each taxon were equal, as determined by a Levene test for homogeneity of variances, a standard F statistic was used to determine the significance of differences between means. When the data variances were different between groups the Welch statistic was employed. And significance of pairwise differences was assessed post hoc using a Tamhane test. All tests were performed as implemented in the SPSS package (SPSS 13.0 for Windows; SPSS, Chicago, Illinois, USA).

### Pollen viability test and hand pollination experiments

Pollen stainability is the common methodology for detecting pollen viability. In the present study we use stainability of pollen with 2, 5-diphenyl tetrazolium bromide (MTT) as an indication of pollen viability [30]. MTT is a vital dye that detects the presence of dehydrogenase by the indication of purple color change for viable pollen grains [31], and the best condition for checking the viability of *Buddleja* using 0.1 % MTT in 20 % sugar solution had been detailed described by Gong [13]. Fresh pollen grains from 4 individuals of each parental species were collected from just-dehisced anthers during the blooming period, and stained with MTT to assess the viability. Then photographs were taken and the percentage of viable (stained) pollen was then calculated. Data was examined for normal distribution with a one sample Kolmogorov Smirnov test. For paired-comparisons between treatments, independent-samples *t* test was included. Data analysis was performed using SPSS 15.0 for Windows (SPSS, Chicago, IL, USA).

Hand pollination experiments were carried out in March 2013 in KBG where *B. crispa* and putative hybrids from Xishan Mountain were successfully introduced several years before. It would be desirable to conduct the pollination experiment in the study area, however it is impossible and not permitted to mark a label and bagging flowers for each sampling tree in such a scenic area with so many tourists each day. Three flowering plants of each taxon were selected to carry out the pollination experiments. For each of the conspecific pollinations the pollen of two of the plants in the same group was mixed to pollinate the third. For heterospecific pollinations the pollen of all three plants in one group was mixed to pollinate each of the six individuals not in the group. For each of the three possible cross-pollinations (one within the group, one from each of the other two groups), 8–15 flowers were randomly selected from each flowering plant. Emasculated flowers were hand-pollinated, and then bagged. In some cases pre-allocated flowers were accidentally damaged before the process was completed, resulting in 20 to 45 successfully pollinated flowers for each of the cross-pollinations. In May 2013, fruits were harvested, and seed numbers were counted.

Seed viability tests were carried out using an X-ray image system (MX-20-DC12, Faxitron, USA, [28]). It should be noted that seed numbers were counted after seed dispersal, as fruit ripeness is difficult to assess in *Buddleja*. Seeds of *Buddleja* taxa in this study were sometimes dispersed when fruits were still green.

### PCR amplification and sequencing of four low-copy genes and nrETS

Total genomic DNA was extracted from silica-dried leaves using a modified CTAB method [32, 33]. The standard protocol was changed as follows: Dried leaves were ground to a fine powder in a Tissue Lyser (Qiagen), and no liquid nitrogen was used; PVP (Polyvinylpyrrolidone) was added to the CTAB extraction buffer. Sequences were obtained for four nuclear loci (gapC1, gapC2, fl1 and defA) and the external transcribed spacer of nuclear ribosomal DNA (nrETS). To design primers for these regions we first used eight pairs of universal primers for angiosperms. Of these primers only the primers for gapC worked for amplification in *Buddleja* [34]. These primers amplified two fragments of different length, which, based on sequence homology, turned out to be members of the gapC gene family. The two regions were therefore designated as gapC1 and gapC2, and two pairs of specific primers were designed for them. We then searched the GenBank for nuclear genes of the genus *Buddleja* and found sequences of three nuclear genes. Based on sequences of fl1 (Floricaula/Leafy-like protein 1, accession number DQ196438) and defA (a MADS box transcription factor of *Buddleja davidii*, accession number HQ853377), we designed primers for the two loci. The nrETS region was amplified using the universal primers ETS and 18S-IGS [35]. Sequences of all used primers are listed in Additional file 1: Table S3.

PCR was conducted using LA Taq DNA polymerase (Takara, Dalian, China) with the following conditions: initial denaturation at 94 °C for 4 min, followed by 30 cycles of 94 °C for 40 s, 53 °C (nrETS, gapC1 and gapC2) or 52 °C (defA and fl1) for 45 s, and 72 °C for 75 s; finishing with a final extension at 72 °C for 10 min. The PCR products were purified by running them on a 1.2 % agarose gel, followed by extraction using a Pearl Gel Extraction Kit (Pearl Biotech, Guangzhou, China), and were then directly sequenced on an ABI 3730 DNA Analyzer using the BigDye Terminator Cycle Sequencing Ready Reaction Kit (Applied Biosystems, Foster City, California, USA). Intra-individual length polymorphism for the nuclear genes could cause failure of direct sequencing from the polymorphic sites. In addition, some individuals, mainly from the putative hybrid, had superimposed chromatograms at multiple sites of the nuclear genes, and the haplotypes could not be reliably inferred. Under these circumstances, cloning sequencing was used to phase the haplotypes. Ligations were conducted using the pMD18-T&A cloning kit (Takara, Dalian, China). Eight positive clones for each individual were selected for sequencing.

### Sequence analyses

Sequences of the four nuclear loci and nrETS regions were aligned and compared in SeqMan™ (DNASTAR, Madison, Wisconsin, USA). As nrETS is generally believed to be homogenized by concerted evolution [36, 37], we treated nrETS, as a single locus, despite the presence of numerous copies in most plant genomes [37]. For *B. crispa* and *B. officinalis*, haplotypes were inferred as implemented by PHASE in DNASP5.0. Haplotype networks were constructed for each locus using Network 4.6.1.0 with the median-joining algorithm [38]. The program NewHybrids was used to assign each individual to a genotype category (parents, F1, F2, backcrosses) using the default settings [39]. This approach does not require that parental populations are sampled separately [39], assuming that only two generations of crossing have occurred. Using this program requires certain assumptions about the markers used: being unlinked, not subject to selection, and at linkage equilibrium in the parental species before hybridization [11]. We thus treated each haplotype as an allele, and conducted linkage disequilibrium test using the program Arlequin ver 3.5.1.3 [40]. Tajima’s neutrality test was conducted for each locus in each parental species in DnaSP v5 [41]. We found no evidence for linkage disequilibrium between these loci in the parental species and for selection at each of these loci.

Genomic admixture proportions of all individuals were assessed using the program Structure version 2.3.1 [42]; the default settings were used, employing the admixture model with correlated allele frequencies. Run parameters were set to 100,000 iterations of MCMC, preceded by a burn-in of 100,000. No prior knowledge of the species was included, and no popflags were set. To determine the most likely number of clusters K, we calculated △K by performing nine runs for each K ranging from 1 to 10 [20].

## Additional files

Additional file 1: Table S1. - The probabilities of each genetic clusters of NewHybrids analysis. **Table S2** - The probabilities of each genetic clusters of Structure analysis. **Table S3** - Sequences of primers used in this study. Additional file 2: Figure S1. Value of △K from the Structure analyses.
