# Supplementary material for: Natural hybridization and asymmetric introgression at the distribution margin of two Buddleja species with a large overlap
Source: BMC Plant Biol. 2015 Jun 18;15:146. doi: 10.1186/s12870-015-0539-9 (PMC4470074; doi:10.1186/s12870-015-0539-9)
Supplement: Additional file 1: Table S1. — - The probabilities of each genetic clusters of NewHybrids analysis. Table S2 - The probabilities of each genetic clusters of Structure analysis. Table S3 - Sequences of primers used in this study. [file 12870_2015_539_MOESM1_ESM.doc]

***Table S1*** *Sequences of primers used in this study*

| Gene or intergenic spacer | Primer name | Primer sequences (5’-3’) |
| --- | --- | --- |
| nrETS | ETS | CAG CGA CAA CAT CCT CTT CA |
|  | 18S-IGS | TCG AGT CCT CGT ACC CAC TT |
| gapC1 | GapC1_F | CTC CAT CAC TGC CAC TCA AA |
|  | GapC1_R | TGA TCT GCA CAA AGG CAA AG |
| gapC2 | GapC2_F | AAG AGC CGC TTC GTT CAA TA |
|  | GapC2_R | GAC CTG CGG ATT CAA CAT TT |
| fl1 | Fl1_R | CAC GGC ATT GCT CAT AGA GA |
|  | Fl1-M | AAT GAA AAC TGT GAA GTA GGG TTG |
| defA | defA-R | AGC CTC AGA GAA CTA TCC ACA |
|  | defA-M1 | TCG AAA CAA ACT TCG ATT TGA TC |
|  | defA-M2 (for sequencing) | TCG AAA CGA ACT TTG ATT TGA TC |

**Table S2** The probabilities of each genetic clusters of NewHybrids analysis

| individual | *B. crispa* | *B. officinalis* | F1 generation | F2 generation | Back cross to *B.crispa* | Back cross to *B. officinalis* |
| --- | --- | --- | --- | --- | --- | --- |
| M1 | 0 | 0.99731 | 0 | 0.00058 | 0 | 0.00211 |
| M2 | 0 | 0.97662 | 0 | 0.00546 | 0 | 0.01792 |
| M3 | 0 | 0.99950 | 0 | 0.00006 | 0 | 0.00044 |
| M4 | 0 | 0.99950 | 0 | 0.00006 | 0 | 0.00044 |
| M5 | 0 | 0.99949 | 0 | 0.00009 | 0 | 0.00042 |
| M6 | 0 | 0.99937 | 0 | 0.00008 | 0 | 0.00055 |
| M7 | 0 | 0.99694 | 0 | 0.00067 | 0 | 0.00239 |
| M8 | 0 | 0.99951 | 0 | 0.00005 | 0 | 0.00044 |
| M9 | 0 | 0.99949 | 0 | 0.00005 | 0 | 0.00046 |
| M10 | 0 | 0.99907 | 0 | 0.00034 | 0 | 0.00059 |
| M11 | 0 | 0.99951 | 0 | 0.00005 | 0 | 0.00044 |
| M12 | 0 | 0.99951 | 0 | 0.00005 | 0 | 0.00044 |
| M13 | 0 | 0.99951 | 0 | 0.00005 | 0 | 0.00044 |
| M14 | 0 | 0.99949 | 0 | 0.00005 | 0 | 0.00046 |
| M15 | 0 | 0.99938 | 0 | 0.00008 | 0 | 0.00055 |
| M16 | 0 | 0.99949 | 0 | 0.00005 | 0 | 0.00046 |
| M17 | 0 | 0.99921 | 0 | 0.00036 | 0 | 0.00043 |
| M18 | 0 | 0.99949 | 0 | 0.00009 | 0 | 0.00042 |
| M19 | 0 | 0.99946 | 0 | 0.00007 | 0 | 0.00047 |
| M20 | 0 | 0.99950 | 0 | 0.00005 | 0 | 0.00044 |
| P1 | 0 | 0.00260 | 0.99274 | 0.00191 | 0.00235 | 0.00040 |
| P2 | 0 | 0.00268 | 0.99493 | 0.00110 | 0.00090 | 0.00039 |
| P3 | 0 | 0.00268 | 0.99493 | 0.00110 | 0.00090 | 0.00039 |
| P4 | 0 | 0.00266 | 0.97525 | 0.01274 | 0.00080 | 0.00855 |
| P5 | 0 | 0.00251 | 0.99510 | 0.00110 | 0.00091 | 0.00039 |
| P6 | 0 | 0.00251 | 0.99510 | 0.00110 | 0.00091 | 0.00039 |
| P7 | 0 | 0.00263 | 0.99489 | 0.00114 | 0.00090 | 0.00043 |
| P8 | 0 | 0.00263 | 0.99489 | 0.00114 | 0.00090 | 0.00043 |
| P9 | 0 | 0.00268 | 0.99493 | 0.00110 | 0.00090 | 0.00039 |
| P10 | 0 | 0.00271 | 0.99397 | 0.00167 | 0.00090 | 0.00076 |
| P11 | 0 | 0.00263 | 0.99489 | 0.00114 | 0.00090 | 0.00043 |
| P12 | 0 | 0.00263 | 0.99489 | 0.00114 | 0.00090 | 0.00043 |
| P13 | 0 | 0.00257 | 0.96886 | 0.01064 | 0.01753 | 0.00040 |
| P14 | 0 | 0.00263 | 0.99489 | 0.00114 | 0.00090 | 0.00043 |
| P15 | 0 | 0.00267 | 0.99441 | 0.00136 | 0.00096 | 0.00061 |
| P16 | 0 | 0.00268 | 0.99451 | 0.00130 | 0.00090 | 0.00060 |
| P17 | 0 | 0.00257 | 0.99485 | 0.00118 | 0.00095 | 0.00044 |
| P18 | 0 | 0.94890 | 0 | 0.01286 | 0 | 0.03824 |
| P19 | 0 | 0.00257 | 0.99485 | 0.00118 | 0.00095 | 0.00044 |
| P20 | 0.99133 | 0.00232 | 0.00006 | 0.00122 | 0.00503 | 0.00005 |
| P21 | 0 | 0.00263 | 0.99489 | 0.00114 | 0.00090 | 0.00043 |
| P22 | 0 | 0.00267 | 0.99467 | 0.00125 | 0.00081 | 0.00061 |
| P23 | 0 | 0.00228 | 0.99499 | 0.00125 | 0.00101 | 0.00047 |
| Z1 | 0.99631 | 0.00236 | 0.00002 | 0.00015 | 0.00115 | 0.00001 |
| Z2 | 0.99666 | 0.00234 | 0.00002 | 0.00006 | 0.00091 | 0.00002 |
| Z3 | 0.99666 | 0.00234 | 0.00002 | 0.00006 | 0.00091 | 0.00002 |
| Z4 | 0.99655 | 0.00238 | 0.00001 | 0.00010 | 0.00093 | 0.00001 |
| Z5 | 0.99666 | 0.00234 | 0.00002 | 0.00006 | 0.00091 | 0.00002 |
| Z6 | 0.68541 | 0.00239 | 0 | 0.28261 | 0.02936 | 0.00022 |
| Z7 | 0.99652 | 0.00248 | 0 | 0.00010 | 0.00088 | 0.00001 |
| Z8 | 0.99657 | 0.00236 | 0 | 0.00013 | 0.00092 | 0.00002 |
| Z9 | 0.98569 | 0.00249 | 0.00003 | 0.00100 | 0.01078 | 0.00001 |
| Z10 | 0.99634 | 0.00238 | 0.00001 | 0.00014 | 0.00110 | 0.00004 |
| Z11 | 0.99659 | 0.00242 | 0 | 0.00014 | 0.00084 | 0.00001 |
| Z12 | 0.99667 | 0.00221 | 0.00001 | 0.00007 | 0.00102 | 0.00002 |
| Z13 | 0.00024 | 0.00216 | 0.71814 | 0.10443 | 0.17465 | 0.00037 |
| Z14 | 0.99676 | 0.00207 | 0 | 0.00008 | 0.00099 | 0.00010 |
| Z15 | 0.00002 | 0.00224 | 0.90437 | 0.03574 | 0.05722 | 0.00041 |
| Z16 | 0.99694 | 0.00200 | 0 | 0.00008 | 0.00095 | 0.00003 |
| Z17 | 0.99682 | 0.00215 | 0 | 0.00007 | 0.00094 | 0.00003 |
| Z18 | 0.99679 | 0.00150 | 0.00004 | 0.00014 | 0.00098 | 0.00055 |
| Z19 | 0.99687 | 0.00148 | 0.00001 | 0.00018 | 0.00104 | 0.00042 |
| Z20 | 0.98265 | 0.00203 | 0.00020 | 0.00170 | 0.01329 | 0.00014 |
| Z21 | 0.98604 | 0 | 0.00003 | 0.00166 | 0.01226 | 0 |
| Z22 | 0 | 0.00218 | 0.99521 | 0.00121 | 0.00087 | 0.00053 |
| Z23 | 0.99827 | 0 | 0 | 0.00030 | 0.00142 | 0.00001 |
| Z24 | 0.99763 | 0.00002 | 0 | 0.00034 | 0.00199 | 0.00002 |

**Table S3** The probabilities of each genetic clusters of Structure analysis

| individual | Cluster1 | | | | | | | | | | Cluster2 | | | | | | | | | |
| --- | --- | --- | --- | --- | --- | --- | --- | --- | --- | --- | --- | --- | --- | --- | --- | --- | --- | --- | --- | --- |
| M1 | 0.002 | 0.002 | 0.002 | 0.002 | 0.002 | 0.002 | 0.002 | 0.002 | 0.002 | 0.002 | 0.998 | 0.998 | 0.998 | 0.998 | 0.998 | 0.998 | 0.998 | 0.998 | 0.998 | 0.998 |
| M2 | 0.110 | 0.110 | 0.110 | 0.110 | 0.110 | 0.110 | 0.110 | 0.110 | 0.110 | 0.110 | 0.890 | 0.890 | 0.890 | 0.890 | 0.890 | 0.890 | 0.890 | 0.890 | 0.890 | 0.890 |
| M3 | 0.001 | 0.001 | 0.001 | 0.001 | 0.001 | 0.001 | 0.001 | 0.001 | 0.001 | 0.001 | 0.999 | 0.999 | 0.999 | 0.999 | 0.999 | 0.999 | 0.999 | 0.999 | 0.999 | 0.999 |
| M4 | 0.001 | 0.001 | 0.001 | 0.001 | 0.001 | 0.001 | 0.001 | 0.001 | 0.001 | 0.001 | 0.999 | 0.999 | 0.999 | 0.999 | 0.999 | 0.999 | 0.999 | 0.999 | 0.999 | 0.999 |
| M5 | 0.001 | 0.001 | 0.001 | 0.001 | 0.001 | 0.001 | 0.001 | 0.001 | 0.001 | 0.001 | 0.999 | 0.999 | 0.999 | 0.999 | 0.999 | 0.999 | 0.999 | 0.999 | 0.999 | 0.999 |
| M6 | 0.001 | 0.001 | 0.001 | 0.001 | 0.001 | 0.001 | 0.001 | 0.001 | 0.001 | 0.001 | 0.999 | 0.999 | 0.999 | 0.999 | 0.999 | 0.999 | 0.999 | 0.999 | 0.999 | 0.999 |
| M7 | 0.001 | 0.001 | 0.001 | 0.001 | 0.001 | 0.001 | 0.001 | 0.001 | 0.001 | 0.001 | 0.999 | 0.999 | 0.999 | 0.999 | 0.999 | 0.999 | 0.999 | 0.999 | 0.999 | 0.999 |
| M8 | 0.001 | 0.001 | 0.001 | 0.001 | 0.001 | 0.001 | 0.001 | 0.001 | 0.001 | 0.001 | 0.999 | 0.999 | 0.999 | 0.999 | 0.999 | 0.999 | 0.999 | 0.999 | 0.999 | 0.999 |
| M9 | 0.001 | 0.001 | 0.001 | 0.001 | 0.001 | 0.001 | 0.001 | 0.001 | 0.001 | 0.001 | 0.999 | 0.999 | 0.999 | 0.999 | 0.999 | 0.999 | 0.999 | 0.999 | 0.999 | 0.999 |
| M10 | 0.003 | 0.003 | 0.003 | 0.003 | 0.003 | 0.003 | 0.003 | 0.003 | 0.004 | 0.003 | 0.997 | 0.997 | 0.997 | 0.997 | 0.997 | 0.997 | 0.997 | 0.997 | 0.996 | 0.997 |
| M11 | 0.001 | 0.001 | 0.001 | 0.001 | 0.001 | 0.001 | 0.001 | 0.001 | 0.001 | 0.001 | 0.999 | 0.999 | 0.999 | 0.999 | 0.999 | 0.999 | 0.999 | 0.999 | 0.999 | 0.999 |
| M12 | 0.001 | 0.001 | 0.001 | 0.001 | 0.001 | 0.001 | 0.001 | 0.001 | 0.001 | 0.001 | 0.999 | 0.999 | 0.999 | 0.999 | 0.999 | 0.999 | 0.999 | 0.999 | 0.999 | 0.999 |
| M13 | 0.001 | 0.001 | 0.001 | 0.001 | 0.001 | 0.001 | 0.001 | 0.001 | 0.001 | 0.001 | 0.999 | 0.999 | 0.999 | 0.999 | 0.999 | 0.999 | 0.999 | 0.999 | 0.999 | 0.999 |
| M14 | 0.001 | 0.001 | 0.001 | 0.001 | 0.001 | 0.001 | 0.001 | 0.001 | 0.001 | 0.001 | 0.999 | 0.999 | 0.999 | 0.999 | 0.999 | 0.999 | 0.999 | 0.999 | 0.999 | 0.999 |
| M15 | 0.006 | 0.006 | 0.006 | 0.006 | 0.006 | 0.006 | 0.006 | 0.006 | 0.006 | 0.006 | 0.994 | 0.994 | 0.994 | 0.994 | 0.994 | 0.994 | 0.994 | 0.994 | 0.994 | 0.994 |
| M16 | 0.001 | 0.001 | 0.001 | 0.001 | 0.001 | 0.001 | 0.001 | 0.001 | 0.001 | 0.001 | 0.999 | 0.999 | 0.999 | 0.999 | 0.999 | 0.999 | 0.999 | 0.999 | 0.999 | 0.999 |
| M17 | 0.001 | 0.001 | 0.001 | 0.001 | 0.001 | 0.001 | 0.001 | 0.001 | 0.001 | 0.001 | 0.999 | 0.999 | 0.999 | 0.999 | 0.999 | 0.999 | 0.999 | 0.999 | 0.999 | 0.999 |
| M18 | 0.001 | 0.001 | 0.001 | 0.001 | 0.001 | 0.001 | 0.001 | 0.001 | 0.001 | 0.001 | 0.999 | 0.999 | 0.999 | 0.999 | 0.999 | 0.999 | 0.999 | 0.999 | 0.999 | 0.999 |
| M19 | 0.003 | 0.003 | 0.003 | 0.003 | 0.003 | 0.003 | 0.003 | 0.002 | 0.003 | 0.003 | 0.997 | 0.997 | 0.997 | 0.997 | 0.997 | 0.997 | 0.997 | 0.998 | 0.997 | 0.997 |
| M20 | 0.001 | 0.001 | 0.001 | 0.001 | 0.001 | 0.001 | 0.001 | 0.001 | 0.001 | 0.001 | 0.999 | 0.999 | 0.999 | 0.999 | 0.999 | 0.999 | 0.999 | 0.999 | 0.999 | 0.999 |
| P1 | 0.508 | 0.508 | 0.508 | 0.508 | 0.507 | 0.507 | 0.508 | 0.507 | 0.508 | 0.508 | 0.492 | 0.492 | 0.492 | 0.492 | 0.493 | 0.493 | 0.492 | 0.493 | 0.492 | 0.492 |
| P2 | 0.495 | 0.495 | 0.495 | 0.497 | 0.495 | 0.495 | 0.496 | 0.495 | 0.496 | 0.495 | 0.505 | 0.505 | 0.505 | 0.503 | 0.505 | 0.505 | 0.504 | 0.505 | 0.504 | 0.505 |
| P3 | 0.495 | 0.495 | 0.495 | 0.495 | 0.495 | 0.495 | 0.496 | 0.495 | 0.495 | 0.495 | 0.505 | 0.505 | 0.505 | 0.505 | 0.505 | 0.505 | 0.504 | 0.505 | 0.505 | 0.505 |
| P4 | 0.512 | 0.511 | 0.512 | 0.513 | 0.512 | 0.512 | 0.512 | 0.511 | 0.512 | 0.512 | 0.488 | 0.489 | 0.488 | 0.487 | 0.488 | 0.488 | 0.488 | 0.489 | 0.488 | 0.488 |
| P5 | 0.486 | 0.486 | 0.486 | 0.486 | 0.486 | 0.485 | 0.486 | 0.485 | 0.485 | 0.486 | 0.514 | 0.514 | 0.514 | 0.514 | 0.514 | 0.515 | 0.514 | 0.515 | 0.515 | 0.514 |
| P6 | 0.485 | 0.486 | 0.486 | 0.486 | 0.486 | 0.486 | 0.486 | 0.486 | 0.485 | 0.486 | 0.515 | 0.514 | 0.514 | 0.514 | 0.514 | 0.514 | 0.514 | 0.514 | 0.515 | 0.514 |
| P7 | 0.524 | 0.524 | 0.525 | 0.525 | 0.524 | 0.524 | 0.525 | 0.524 | 0.525 | 0.524 | 0.476 | 0.476 | 0.475 | 0.475 | 0.476 | 0.476 | 0.475 | 0.476 | 0.475 | 0.476 |
| P8 | 0.525 | 0.525 | 0.524 | 0.525 | 0.524 | 0.525 | 0.525 | 0.524 | 0.525 | 0.525 | 0.475 | 0.475 | 0.476 | 0.475 | 0.476 | 0.475 | 0.475 | 0.476 | 0.475 | 0.475 |
| P9 | 0.495 | 0.495 | 0.495 | 0.496 | 0.496 | 0.495 | 0.495 | 0.495 | 0.495 | 0.495 | 0.505 | 0.505 | 0.505 | 0.504 | 0.504 | 0.505 | 0.505 | 0.505 | 0.505 | 0.505 |
| P10 | 0.497 | 0.498 | 0.498 | 0.498 | 0.498 | 0.497 | 0.499 | 0.498 | 0.498 | 0.498 | 0.503 | 0.502 | 0.502 | 0.502 | 0.502 | 0.503 | 0.501 | 0.502 | 0.502 | 0.502 |
| P11 | 0.524 | 0.525 | 0.524 | 0.525 | 0.524 | 0.525 | 0.525 | 0.524 | 0.525 | 0.525 | 0.476 | 0.475 | 0.476 | 0.475 | 0.476 | 0.475 | 0.475 | 0.476 | 0.475 | 0.475 |
| P12 | 0.525 | 0.524 | 0.525 | 0.524 | 0.525 | 0.524 | 0.525 | 0.524 | 0.525 | 0.525 | 0.475 | 0.476 | 0.475 | 0.476 | 0.475 | 0.476 | 0.475 | 0.476 | 0.475 | 0.475 |
| P13 | 0.607 | 0.607 | 0.608 | 0.607 | 0.607 | 0.607 | 0.607 | 0.607 | 0.607 | 0.607 | 0.393 | 0.393 | 0.392 | 0.393 | 0.393 | 0.393 | 0.393 | 0.393 | 0.393 | 0.393 |
| P14 | 0.524 | 0.524 | 0.525 | 0.525 | 0.525 | 0.524 | 0.525 | 0.525 | 0.525 | 0.525 | 0.476 | 0.476 | 0.475 | 0.475 | 0.475 | 0.476 | 0.475 | 0.475 | 0.475 | 0.475 |
| P15 | 0.498 | 0.498 | 0.497 | 0.498 | 0.497 | 0.498 | 0.499 | 0.498 | 0.498 | 0.498 | 0.502 | 0.502 | 0.503 | 0.502 | 0.503 | 0.502 | 0.501 | 0.502 | 0.502 | 0.502 |
| P16 | 0.494 | 0.495 | 0.495 | 0.495 | 0.495 | 0.494 | 0.495 | 0.495 | 0.495 | 0.495 | 0.506 | 0.505 | 0.505 | 0.505 | 0.505 | 0.506 | 0.505 | 0.505 | 0.505 | 0.505 |
| P17 | 0.538 | 0.537 | 0.538 | 0.538 | 0.537 | 0.537 | 0.538 | 0.537 | 0.537 | 0.537 | 0.462 | 0.463 | 0.462 | 0.462 | 0.463 | 0.463 | 0.462 | 0.463 | 0.463 | 0.463 |
| P18 | 0.053 | 0.053 | 0.053 | 0.053 | 0.053 | 0.053 | 0.053 | 0.053 | 0.053 | 0.053 | 0.947 | 0.947 | 0.947 | 0.947 | 0.947 | 0.947 | 0.947 | 0.947 | 0.947 | 0.947 |
| P19 | 0.537 | 0.537 | 0.538 | 0.538 | 0.537 | 0.537 | 0.537 | 0.537 | 0.537 | 0.537 | 0.463 | 0.463 | 0.462 | 0.462 | 0.463 | 0.463 | 0.463 | 0.463 | 0.463 | 0.463 |
| P20 | 0.887 | 0.887 | 0.887 | 0.888 | 0.887 | 0.887 | 0.888 | 0.887 | 0.887 | 0.887 | 0.113 | 0.113 | 0.113 | 0.112 | 0.113 | 0.113 | 0.112 | 0.113 | 0.113 | 0.113 |
| P21 | 0.514 | 0.513 | 0.513 | 0.514 | 0.513 | 0.513 | 0.514 | 0.513 | 0.513 | 0.513 | 0.486 | 0.487 | 0.487 | 0.486 | 0.487 | 0.487 | 0.486 | 0.487 | 0.487 | 0.487 |
| P22 | 0.493 | 0.493 | 0.493 | 0.494 | 0.493 | 0.493 | 0.493 | 0.493 | 0.493 | 0.493 | 0.507 | 0.507 | 0.507 | 0.506 | 0.507 | 0.507 | 0.507 | 0.507 | 0.507 | 0.507 |
| P23 | 0.478 | 0.479 | 0.479 | 0.478 | 0.479 | 0.478 | 0.479 | 0.478 | 0.479 | 0.479 | 0.522 | 0.521 | 0.521 | 0.522 | 0.521 | 0.522 | 0.521 | 0.522 | 0.521 | 0.521 |
| Z1 | 0.999 | 0.999 | 0.999 | 0.999 | 0.999 | 0.999 | 0.999 | 0.999 | 0.999 | 0.999 | 0.001 | 0.001 | 0.001 | 0.001 | 0.001 | 0.001 | 0.001 | 0.001 | 0.001 | 0.001 |
| Z2 | 0.999 | 0.999 | 0.999 | 0.999 | 0.999 | 0.999 | 0.999 | 0.999 | 0.999 | 0.999 | 0.001 | 0.001 | 0.001 | 0.001 | 0.001 | 0.001 | 0.001 | 0.001 | 0.001 | 0.001 |
| Z3 | 0.999 | 0.999 | 0.999 | 0.999 | 0.999 | 0.999 | 0.999 | 0.999 | 0.999 | 0.999 | 0.001 | 0.001 | 0.001 | 0.001 | 0.001 | 0.001 | 0.001 | 0.001 | 0.001 | 0.001 |
| Z4 | 0.999 | 0.999 | 0.999 | 0.999 | 0.999 | 0.999 | 0.999 | 0.999 | 0.999 | 0.999 | 0.001 | 0.001 | 0.001 | 0.001 | 0.001 | 0.001 | 0.001 | 0.001 | 0.001 | 0.001 |
| Z5 | 0.999 | 0.999 | 0.999 | 0.999 | 0.999 | 0.999 | 0.999 | 0.999 | 0.999 | 0.999 | 0.001 | 0.001 | 0.001 | 0.001 | 0.001 | 0.001 | 0.001 | 0.001 | 0.001 | 0.001 |
| Z6 | 0.884 | 0.888 | 0.888 | 0.889 | 0.885 | 0.887 | 0.891 | 0.885 | 0.888 | 0.887 | 0.116 | 0.112 | 0.112 | 0.111 | 0.115 | 0.113 | 0.109 | 0.115 | 0.112 | 0.113 |
| Z7 | 0.999 | 0.999 | 0.999 | 0.999 | 0.999 | 0.999 | 0.999 | 0.999 | 0.999 | 0.999 | 0.001 | 0.001 | 0.001 | 0.001 | 0.001 | 0.001 | 0.001 | 0.001 | 0.001 | 0.001 |
| Z8 | 0.999 | 0.999 | 0.999 | 0.999 | 0.999 | 0.999 | 0.999 | 0.999 | 0.999 | 0.999 | 0.001 | 0.001 | 0.001 | 0.001 | 0.001 | 0.001 | 0.001 | 0.001 | 0.001 | 0.001 |
| Z9 | 0.931 | 0.932 | 0.932 | 0.932 | 0.932 | 0.932 | 0.932 | 0.931 | 0.932 | 0.932 | 0.069 | 0.068 | 0.068 | 0.068 | 0.068 | 0.068 | 0.068 | 0.069 | 0.068 | 0.068 |
| Z10 | 0.999 | 0.999 | 0.999 | 0.999 | 0.999 | 0.999 | 0.999 | 0.999 | 0.999 | 0.999 | 0.001 | 0.001 | 0.001 | 0.001 | 0.001 | 0.001 | 0.001 | 0.001 | 0.001 | 0.001 |
| Z11 | 0.999 | 0.999 | 0.999 | 0.999 | 0.999 | 0.999 | 0.999 | 0.999 | 0.999 | 0.999 | 0.001 | 0.001 | 0.001 | 0.001 | 0.001 | 0.001 | 0.001 | 0.001 | 0.001 | 0.001 |
| Z12 | 0.999 | 0.999 | 0.999 | 0.999 | 0.999 | 0.999 | 0.999 | 0.998 | 0.999 | 0.999 | 0.001 | 0.001 | 0.001 | 0.001 | 0.001 | 0.001 | 0.001 | 0.002 | 0.001 | 0.001 |
| Z13 | 0.548 | 0.549 | 0.548 | 0.548 | 0.548 | 0.548 | 0.548 | 0.549 | 0.548 | 0.548 | 0.452 | 0.451 | 0.452 | 0.452 | 0.452 | 0.452 | 0.452 | 0.451 | 0.452 | 0.452 |
| Z14 | 0.949 | 0.951 | 0.952 | 0.953 | 0.949 | 0.951 | 0.954 | 0.951 | 0.952 | 0.951 | 0.051 | 0.049 | 0.048 | 0.047 | 0.051 | 0.049 | 0.046 | 0.049 | 0.048 | 0.049 |
| Z15 | 0.550 | 0.551 | 0.550 | 0.551 | 0.550 | 0.550 | 0.551 | 0.550 | 0.551 | 0.550 | 0.450 | 0.449 | 0.450 | 0.449 | 0.450 | 0.450 | 0.449 | 0.450 | 0.449 | 0.450 |
| Z16 | 0.997 | 0.997 | 0.997 | 0.997 | 0.997 | 0.997 | 0.997 | 0.997 | 0.997 | 0.997 | 0.003 | 0.003 | 0.003 | 0.003 | 0.003 | 0.003 | 0.003 | 0.003 | 0.003 | 0.003 |
| Z17 | 0.867 | 0.869 | 0.869 | 0.872 | 0.866 | 0.870 | 0.873 | 0.868 | 0.870 | 0.869 | 0.133 | 0.131 | 0.131 | 0.128 | 0.134 | 0.130 | 0.127 | 0.132 | 0.130 | 0.131 |
| Z18 | 0.948 | 0.950 | 0.950 | 0.951 | 0.947 | 0.949 | 0.951 | 0.948 | 0.950 | 0.949 | 0.052 | 0.050 | 0.050 | 0.049 | 0.053 | 0.051 | 0.049 | 0.052 | 0.050 | 0.051 |
| Z19 | 0.999 | 0.999 | 0.999 | 0.999 | 0.999 | 0.999 | 0.999 | 0.999 | 0.999 | 0.999 | 0.001 | 0.001 | 0.001 | 0.001 | 0.001 | 0.001 | 0.001 | 0.001 | 0.001 | 0.001 |
| Z20 | 0.919 | 0.919 | 0.919 | 0.919 | 0.919 | 0.919 | 0.919 | 0.919 | 0.919 | 0.919 | 0.081 | 0.081 | 0.081 | 0.081 | 0.081 | 0.081 | 0.081 | 0.081 | 0.081 | 0.081 |
| Z21 | 0.934 | 0.934 | 0.934 | 0.934 | 0.934 | 0.934 | 0.934 | 0.934 | 0.934 | 0.934 | 0.066 | 0.066 | 0.066 | 0.066 | 0.066 | 0.066 | 0.066 | 0.066 | 0.066 | 0.066 |
| Z22 | 0.405 | 0.406 | 0.406 | 0.406 | 0.405 | 0.406 | 0.406 | 0.406 | 0.406 | 0.406 | 0.595 | 0.594 | 0.594 | 0.594 | 0.595 | 0.594 | 0.594 | 0.594 | 0.594 | 0.594 |
| Z23 | 0.999 | 0.999 | 0.999 | 0.999 | 0.999 | 0.999 | 0.999 | 0.999 | 0.999 | 0.999 | 0.001 | 0.001 | 0.001 | 0.001 | 0.001 | 0.001 | 0.001 | 0.001 | 0.001 | 0.001 |
| Z24 | 0.999 | 0.999 | 0.999 | 0.999 | 0.999 | 0.999 | 0.999 | 0.999 | 0.999 | 0.999 | 0.001 | 0.001 | 0.001 | 0.001 | 0.001 | 0.001 | 0.001 | 0.001 | 0.001 | 0.001 |


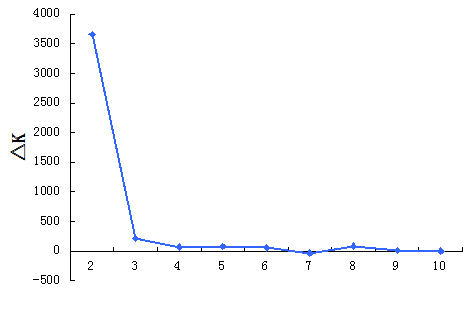


**Fig. S1.** Value of △K from the Structure analyses.
