# Supplementary figures and images for: Natural hybridization and asymmetric introgression at the distribution margin of two Buddleja species with a large overlap
Source: BMC Plant Biol. 2015 Jun 18;15:146. doi: 10.1186/s12870-015-0539-9 (PMC4470074; doi:10.1186/s12870-015-0539-9)

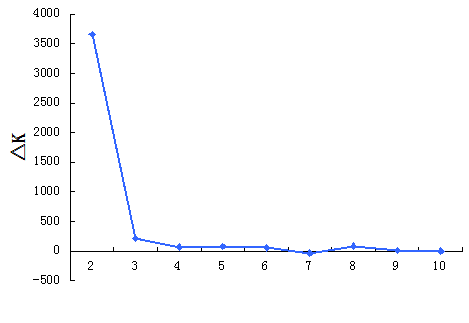

Supplement: Additional file 2: Figure S1. — Value of △K from the Structure analyses. [file 12870_2015_539_MOESM2_ESM.png]
